# Patient-derived intestinal organoids as a model for site-specific mucosal bacterial interactions in paediatric inflammatory bowel disease

**DOI:** 10.1038/s41598-026-46184-8

**Published:** 2026-04-01

**Authors:** Eva Chan, Wing Hei Chan, Genevieve Kerr, Stuart K. Archer, Thierry Jardé, Rebekah M. Engel, Jodee A. Gould, Shanika L. Amarasinghe, Emily L. Rutten, Gemma L. D’Adamo, Emily L. Gulliver, Linden J. Gearing, Samuel C. Forster, Edward M. Giles, Helen E. Abud

**Affiliations:** 1 Department of Anatomy and Developmental Biology, Monash University, Clayton, VIC 3800 Australia; 2 Development and Stem Cells Program, Monash Biomedicine Discovery Institute, Monash University, Clayton, VIC 3800 Australia; 3 Centre for Innate Immunity and Infectious Diseases, Hudson Institute of Medical Research, Clayton, VIC 3168 Australia; 4 Department of Molecular and Translational Sciences, Monash University, Clayton, VIC 3800 Australia; 5 Monash Genomics and Bioinformatics Platform, Monash University, Clayton, VIC 3800 Australia; 6 Department of Surgery, Cabrini Hospital, Cabrini Monash University, Malvern, VIC 3144 Australia; 7 Department of Paediatrics, Monash University, Clayton, VIC 3800 Australia

**Keywords:** Paediatric inflammatory bowel disease, Patient-derived organoids, Bacteria-organoid co-culture, Microinjection, Gastroenterology, Microbiology

## Abstract

Inflammatory bowel disease (IBD) is secondary to an abnormal immune response to the microbiota. To study this, models of host-microbe interactions that represent mucosal bacterial communities and inter-patient diversity are required. Human intestinal organoids (HIOs) are an established model to investigate epithelial responses. Here, we describe a technique of culturing bacteria directly from the sites of inflammation in IBD, while simultaneously sampling host tissue. We generated HIOs from a cohort of newly diagnosed paediatric IBD patients, without confounding treatments or comorbidities, and explored their response to site-specific bacteria. A unique biobank of matched HIOs and cultured mucosa-attached bacteria was established from 27 paediatric patients. Transcriptional profiling revealed differential gene expression between control and IBD-derived organoids. We used microinjection to introduce bacteria to the apical surface of the epithelium, to determine the effect of bacteria on host epithelial cells. We measured survival and growth of bacteria within the HIOs and tested several related bacterial isolates for their impact on the epithelium. An isolate from a control patient stimulated inflammatory signalling pathways but this was not observed in response to a closely related isolate originating from an IBD patient. This study demonstrates the feasibility of isolating bacteria and generating organoids from the same biopsy tissue, to explore personalised host-microbe interactions. The microinjections, while labour-intensive, demonstrate that closely related bacteria can induce very different epithelial responses, with downstream implications for immune response. This highlights the importance of understanding host-microbe interactions in a strain- and site-specific manner and developing techniques for personalised microbiome-based therapeutics.

**Supplementary Information:**

The online version contains supplementary material available at 10.1038/s41598-026-46184-8.

## Introduction

Inflammatory bowel disease (IBD) impacts the lining of the gastrointestinal tract where the intestinal epithelium is the interface of host-microbe interactions^1^. The epithelial monolayer is the first line of defence against the luminal environment, mediating signals between the body’s immune response and the microbiome to preserve the intestinal barrier^1,2^. IBD includes both Crohn’s disease (CD) and ulcerative colitis (UC), where more than 300 risk loci have been identified with IBD^3,4^. Many of these loci have been associated with alterations to the intestinal epithelial barrier^5^, mucosal immunity^6,7^ and disruption to epithelial cell function^7^. Transcriptional alterations have also been observed in epithelial stem and proliferative cell populations in IBD^8,9^, suggesting defects in epithelial repair may contribute to IBD pathology.

Paediatric IBD patients develop more severe disease and substantial lifelong complications impacting growth and absorption of nutrients^10^. Deciphering the mechanism of disease in paediatric patients at diagnosis may provide insight into how IBD initiates and provide an opportunity to intervene to mitigate long-term damage. Disease modelling of IBD requires a system that recapitulates the function and heterogeneity of cells within the human epithelium. As mouse models and immortalised cell lines do not faithfully represent human intestinal tissue, patient-derived intestinal organoid cultures can be used to study epithelial disease phenotypes and response to commensals and pathogens on apical and basolateral surfaces^9,11–13^. Whilst organoid cultures are enriched with stem and proliferative cell types, scRNA-sequencing has identified diversity within these cultures, where goblet cells, early enterocytes, enteroendocrine cells and M-cells are also present^14^.

Regional differences in microbial composition are observed throughout the gastrointestinal tract (GIT)^15,16^ and changes in the microbiome are a key observation in both paediatric and adult patients with IBD^17,18^. Studies have utilised patient faecal samples to decipher the intestinal microbiome community of IBD patients^17,18^. However, faecal microbes do not necessarily represent the mucosa-associated microbial communities in the GIT^19^, where, for example, there are distinct microbial niches identified in the colon^16,20^. Therefore, to better understand direct host-microbial interactions, studies need to include bacteria found at the site-specific mucosal surface of tissues.

With the ability to culture anaerobic bacteria from the human microbiome^21^, including a recently developed technique from biopsies^20^, it is now possible to gain higher-level resolution of the bacterial species in control and IBD patients. In this study, we combine establishment of human intestinal organoids (HIOs) from different regions of the GIT with culturing mucosa-associated bacteria from paediatric control and IBD patients. With the use of microinjection to perform physiologically relevant bacteria-organoid co-cultures, this allows assessment of both patient- and site-specific host-microbe interactions and generates a resource to model epithelial barrier dysfunction in paediatric disease. This demonstrates the culturing capabilities from patient biopsy tissue and the application of microinjection to interrogate host-microbe interactions in a modest cohort size.

## Methods

### Patient recruitment

Ethics approval from the Monash Health Human Research Ethics Committee was granted under HREC/16/MonH/253 and Monash Human Research Ethics Committee (MHREC ID: 9876). Paediatric patients undergoing gastroscopy and colonoscopy were recruited for this study based on presentation of symptoms. Patients with known or suspected monogenic disorders were excluded from recruitment. Informed consent was obtained from all patient’s legal guardian(s). Two biopsy samples were collected for organoid and bacterial culturing from control patients and patients newly diagnosed with IBD, where all experiments were performed in accordance with relevant guidelines and regulations, as a result, no genetic testing was carried out with patient samples.

### Bacterial isolation and culture from patient biopsy samples

Biopsies were collected into sterile microcentrifuge tubes stored on ice until processing. Isolation of bacteria was carried out within 1 h of tissue collection by suspending the tissue in a minimum of 50 µL anaerobic PBS, followed by vortex mixing for 1 min to isolate mucosa adhered bacteria. All bacterial culturing was carried out under anaerobic conditions (80% N_2_, 10% CO_2_, 10% H_2_ at 37 °C) in a Whitley A95 workstation (Don Whitley Scientific). Cultures were plated on pre-reduced yeast casitone fatty acids (YCFA) agar plates and grown overnight.

### Organoid generation and culture

HIOs were cultured from the small intestine (duodenum and terminal ileum), to understand early host-microbe interactions in the proximal bowel. In many of our organoids, this allowed analysis of macroscopically and histologically non-inflamed tissue, especially in the UC cohort. Organoids were cultured as previously described^22^ and as follows. The biopsy tissue was placed into media containing HibernateTM-A Medium (GibcoTM, #A1247501), 1X Penicillin/Streptomycin (GibcoTM, #15140122), 100 µg/mL Primocin (InvivoGen, #ant-pm-2) and 10 µM Y-27,632 dihydrochloride (MedChem Express LLC, #HY-10583). Biopsy tissue was cut into small pieces with sterile scalpel blades and washed in Advanced DMEM/F12 (GibcoTM, #12634010) containing 1X Pen/Strep and centrifuged at 423×*g* for 5 min at 4 °C, twice. Dissociated tissue was resuspended in Matrigel^®^ Growth Factor Reduced Basement Membrane Matrix (Corning^®^, #FAL356231) and seeded in a 24-well plate (Thermo Scientific™, #142475), at 40 µL per well. 500 µL of medium containing Advanced DMEM/F12, 1X GlutaMAX™ (Gibco™, #35050061), 10 mM HEPES (Gibco™, #15630080), 100 µg/mL Primocin (InvivoGen, #ant-pm-2), 1X B-27™ Supplement.

(Gibco™, #17504044), 50% Wnt3a conditioned media (CM), 20% R-spondin1 CM, 5% Noggin CM, 50 ng/mL Human EGF (PeproTech, #AF- 100 − 15), 10 mM Nicotinamide (Sigma-Aldrich, #N0636), 1.25 mM N-Acetyl-L- cysteine (Sigma-Aldrich, #A9165), 10nM Human Gastrin I (Sigma-Aldrich, #G9145), 0.5 µM A83-01 (Tocris, #2939), 100 ng/mL Recombinant Human IGF1 (BioLegend, #590908), 50 ng/mL Recombinant Human FGF Basic (PreproTech, #100-18-500) was overlayed. 2.5 µM Stemolecule Chir99021 (Stemgent, #04–0004) and 10 µM Y-27,632 dihydrochloride (MedChem Express LLC, #HY-10583) was added to the culture medium for the first 2–3 days following biopsy tissue seeding and organoid passaging. Cultures were maintained in 5% CO_2_ at 37 °C and media replaced every second to third day.

All conditioned media was provided by the Monash Biomedicine Discovery Institute Organoid Program.

### Microinjections

Using the Flaming/Brown Micropipetter Puller (Sutter Instrument Company P-87), glass capillaries (Harvard Apparatus #30–0083) were pulled to a fine point with a 5–10 μm opening. Capillaries were loaded with 4 kDa fluorescein isothiocyanate (FITC)- dextran, with or without bacterial sample, or 40 kDa FITC-dextran in 1X PBS for microinjection into cystic organoids. All microinjections were carried out under a Zeiss SteREO Discovery.V8 microscope using the Injectman 4 Micromanipulator coupled with FemtoJet 4i system (Eppendorf). Injection parameters were set at 120 hPa (injection pressure), 0.25 s (injection time) and 0 hPa (compensation pressure). Microinjected organoids were individually picked for RNA extraction. This involved using a p10 pipette tip with the tip cut off to create a larger opening, this was used to pick 45–50 organoids from the surrounding Matrigel, per condition. Picked organoids were pipetted directly into Buffer RLT and pipetted to lyse. Samples were stored at -80 °C prior to RNA isolation. All microinjection experiments were performed as 3 experimental repeats.

### Permeability assay

20–25 cystic organoids were microinjected per condition, with either 4 kDa FITC-dextran (#46944-100MG- F, Sigma-Aldrich), 4 kDa FITC-dextran with bacteria, 4 kDa FITC-dextran with 3 mM EGTA added 1 h prior to endpoint or 40 kDa FITC-dextran (#FD40-100 mg, Sigma- Aldrich) and imaged at time 0 h and 24 h post-injection with ImageXpress Pico Automated Cell Imaging System (Molecular Devices). Brightfield and fluorescent images were captured for analysis. An automated analysis module was created utilising MetaXpress version 6.7.1.157 (Molecular Devices) to measure luminal fluorescence and area of microinjected organoids. The transmitted light image was used to generate an outline of the organoids within the field of view. A mask was generated of the organoids with a minimum width of 100 μm and an intensity above local background of 100 units. This mask image was then used to measure organoid area. The FITC image was used to detect organoids that had been microinjected where a mask was generated of the fluorescent signal with a minimum width of 100 μm and an intensity above local background of 13 units. This mask was then used to identify microinjected organoids at the 0 h timepoint and compared to the 24 h timepoint image, due to limitations with organoid tracking at the 2 timepoints, 2–8 organoids were measured per condition, per experiment.

### RNA extraction, RNA-sequencing and data analysis

Organoids were grown for 8 days post-passage for RNA, where most organoids were collected at an early passage (passage 2). However, some organoids were collected at passage 3 (hSI07, hSI26, hSI27, hSI33, hSI55 and hSI57), passage 4 (hSI08), passage 5 (hSI22, hSI23) and passage 6 (hSI29) for RNA. Total RNA was isolated using the RNeasy Micro Kit (Qiagen, #74004) and assessed for quality using the Bioanalyzer (Agilent) and Qubit (Invitrogen). RNA-sequencing was performed by the Hudson Genomics Facility, Australia, where 20 ng of total RNA was used for library preparation with index sequences added during first strand synthesis. Pooled libraries were sequenced using 19 bp (bp) forward read and 72 bp reverse read on Next Seq 550 V2.5 (human organoids) or 19 bp (bp) forward read and 101 bp reverse read on Next Seq 2000 p3 (microinjected organoids). Reads were de-multiplexed using SABRE version 663365f^23^ and then mapped to the human genome reference, GRCh38, using STAR version 2.7.2b^24^. SAMtools version 1.9^25^ was then used to process read alignments, where duplicated alignments were marked using Picard MarkDuplicates version 2.18.9^26^. Duplicates with Unique Molecule Identifiers (UMIs) were processed with Je markdupes version 2.0RC^27^. The unique alignments were counted in gene features using featureCounts in Rsubread version 1.6.1^28^ resulting in the gene-count matrix. The resulting gene-count matrix was filtered for genes with > 1 CPM in at least 3 samples, and > 5 counts in a minimum of 1 sample. Gene counts were run through EdgeR-quasi-likelihood version 3.32.1^29^. For human organoid RNA-sequencing, data was modelled with 7 hidden factors and for organoid microinjection RNA-sequencing, data was modelled with 5 hidden factors using RUVr version 1.24.0^30^. Genes were further analysed by applying False Discovery Rate (FDR, calculated by the Benjamini-Hochberg procedure) ≤ 0.05, Log_2_ Fold Change (log_2_FC) ≥ 1 or ≤ − 1 for significantly differentially expressed genes. Functional annotation was carried out by Metascape^31^, Benjamini-Hochberg procedure was performed, where q ≤ 0.05 was considered significant.

### Bacterial sequencing and analysis

#### 16S RNA capillary sequencing

Bacterial isolates were picked and identified via 16S RNA capillary sequencing, using broad range 16S rRNA gene primers, forward primer 5’-AGA GTT TGA TYM TGG CTC AG-3 and reverse primer 5’-ACG GYT ACC TTG TTA CGA CTT-3’^20^. Taxonomic classification was performed with BLASTn v2.9^32^ using SILVA 138.1^33^. MAFFT v7.453 was used to align sequences and phylogenetic relationship was performed using RAxML v8.2.11^34^ and visualised with iTOL v6^35^.

### Whole genome sequencing

Bacterial DNA was isolated using the MP Biomedicals FastDNA SPIN Kit for soil, followed by sequencing on Illumina NextSeq2000 or Illumina NextSeq550. Trimmomatic v 0.38^36^ was used to trim sequences, and sequences assembled using SPAdes v 3.13.0^37^. CheckM v 1.1.3 was used for quality control of assembled sequences^38^.

To determine average nucleotide identity (ANI), nucleotide sequences of genomes were compared using dRep compare with the default parameters^39^. Genomes were then annotated using Bakta v 1.8.2^40^ and the resultant gff3 files were used to determine the core- and pan-genomes of the isolate pairs using panaroo v 1.5.0 using the default parameters^41^.

### Bacterial growth and survival assays

Individual bacterial colonies were inoculated into 10 mL pre-reduced YCFA broth and incubated for 24 h in anaerobic conditions (80% N_2_, 10% CO_2_, 10% H_2_ at 37 °C). Once grown in broth, samples were serially diluted 1:10, eight times. 10 µL of sample was spot plated in triplicate, per dilution, per sample. Plates were incubated in either anaerobic, microaerophilic or aerobic conditions for 24 h at 37 °C for assessment of bacteria growth in different conditions. To assess bacterial growth over time in aerobic conditions, inoculated bacterium was diluted 1:1000 in fresh YCFA broth per well of a 96-well plate. Samples were incubated at 37 °C, and measurements taken in the Omega microplate reader (BMG). Each well was measured for 24 cycles, with each cycle being 60 min apart. Prior to a cycle, the plate was shaken for 10 s in a double orbital movement at 300 rpm. The top optic was used to scan a 3 mm diameter area of each well after 31 flashes per well and cycle was carried out. The absorbance of each well was measured at an excitation of 600 nm. To assess bacteria survival, spot plated samples were exposed to aerobic conditions (room temperature conditions) for 1 h, 2 h, 3 h, or 6 h, followed by 24 h incubation in anaerobic conditions. To quantify CFU per microinjection, sample from the capillary was tested throughout the experiment by sampling pre-, mid- and post-microinjection. This involved injecting into 50 µl volumes of 1x PBS, 3 times from the capillary. Sample was pipetted to mix then serially diluted and plated as described for bacterial growth assays. To quantify CFU per organoid, 4 microinjected organoids were picked from the Matrigel using a p10 pipette tip with the tip cut off with a sterile scalpel blade. Picked organoids were placed into 1x PBS and mechanically disrupted by pipetting, then serially diluted and plated as described for bacterial growth assays. To quantify CFU per ml of media, media was collected at 0 h, 2 h, and 24 h, where samples were sample were serially diluted and plated as described for bacterial growth assays. For the 0 h time point, 700 µl media was also streak plated.

### Statistical analysis

Statistical significance (*p* < 0.05) was determined using the statistical tests as outlined in the figure legends. Analysis was carried out in GraphPad Prism (version 10.2.0).

## Results

### A dual isolation protocol enables establishment of patient-derived organoids and mucosa-attached bacteria

**Fig. 1:**
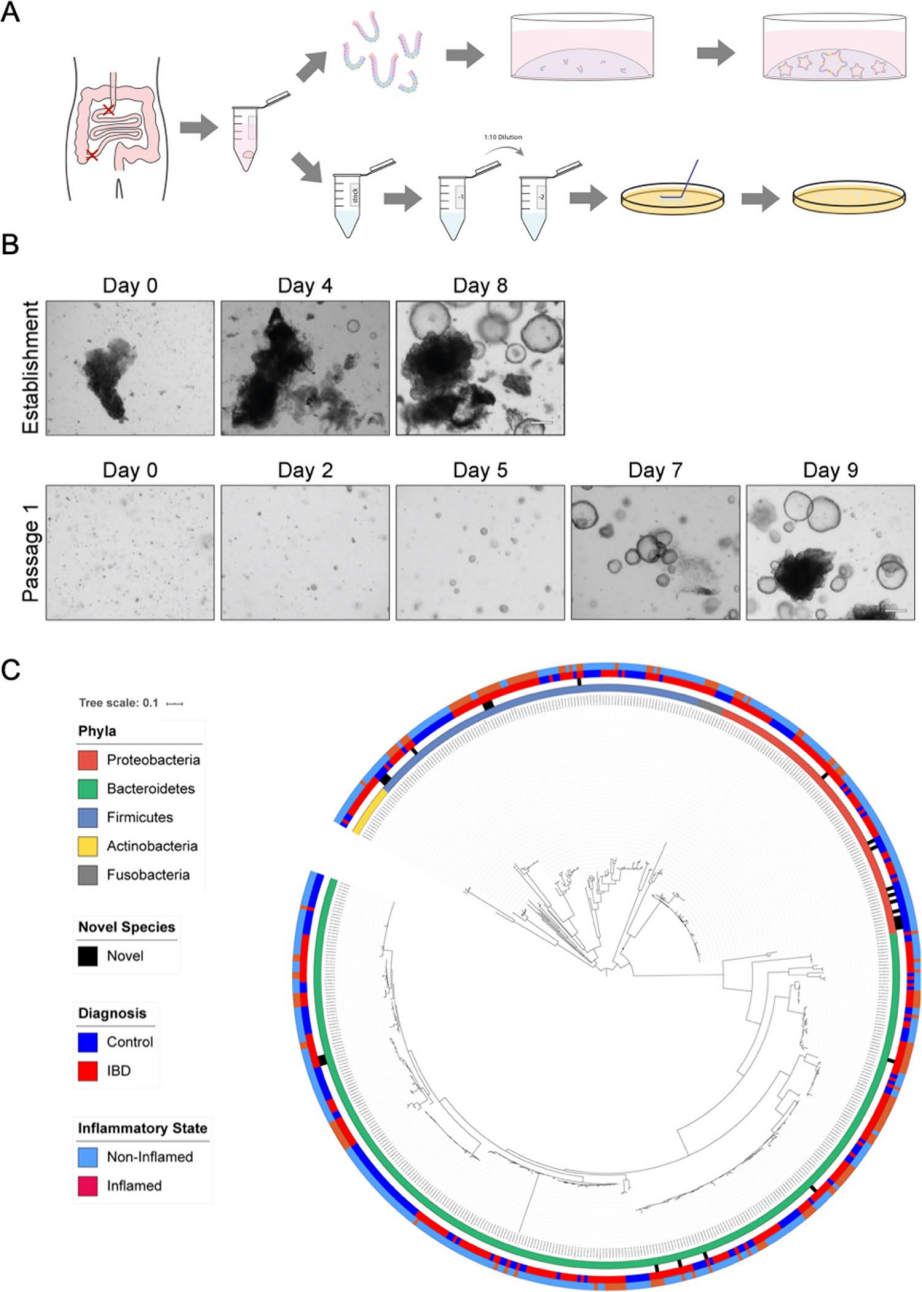
Dual isolation protocol for bacterial culture and establishment of organoids from biopsies. (**A**) Diagrammatic representation of biopsy collection and subsequent culturing procedure. Red crosses indicate site at which patient biopsy samples were taken. (**B**) Representative images of organoids established from biopsy tissue. Prior to embedding of tissue in Matrigel, bacteria were isolated, and tissue mechanically dissociated. 49 organoid lines were established. Scale bar 500 μm. (**C**) Phylogenetic tree of 538 bacterial isolates from patient terminal ileum biopsies. Bacterial isolates are grouped according to phyla (inner-most ring), Pseudomonadota (Proteobacteria) in red (89 isolates), Bacteroidota (Bacteroidetes) in green (320 isolates), Bacillota (Firmicutes) in blue (105 isolates), Actinomycetota (Actinobacteria) in yellow (16 isolates) and Fusobacteria in grey (8 isolates). Putative novel species (27 isolates) are indicated with a black bar (second ring). Patient information regarding diagnosis is overlaid on the tree (third ring), with control patients in blue (210 isolates) and IBD patients in red (328 isolates). Information regarding presence of inflammation at time of diagnosis is also overlayed (outer-most ring), with isolates associated with non-inflamed tissue in blue (394 isolates) and isolates associated with inflamed tissue in red (144 isolates) (outer most ring).

Generating authentic personalised models of human disease is challenging, especially if dependent on small biopsies of tissue. Here, we describe the process for effectively isolating mucosa-attached bacteria from patient biopsy tissue to study site-specific microbial communities and epithelial cells to establish organoids. Mucosa-attached bacteria were isolated by vortex mixing and plating on YCFA plates, in anaerobic conditions^20,21^. Human epithelial cells were then processed for organoid culturing by mechanical dissociation and embedding directly into Matrigel (Fig. 1A). A total of 49 organoid lines were established from duodenum and/or terminal ileum biopsies from 27 patients (Fig. 1B). The terminal ileum was chosen for consistency across all IBD subtypes (and controls) and is a site of intense immune interaction with the microbiome. Additionally, the duodenum and terminal ileum have also been less studied in organoid analyses in IBD than colonic samples. 538 bacterial isolates were cultured from terminal ileum biopsies and the phyla identified with 16 S rRNA capillary sequencing (Supplementary Table 1). Of these isolates, there were 89 Pseudomonadota (Proteobacteria), 105 Bacillota (Firmicutes), 320 Bacteroidota (Bacteroidetes), 16 Actinomycetota (Actinobacteria) and 8 Fusobacteria (Fig. 1C). This methodology was applied to both control tissue, tissue from patients with IBD and tissue with and without active inflammation (Table 1). No bacteria could be cultured from the duodenal biopsies, likely due to the reduced bacteria load.

**Table 1:** Summary of all patients recruited in study.

	IBD		
	Control	CD	UC
Diagnosis	12	11	4
Gender (Female: Male)	8:4	10:1	2:2
Mean Age (years)	12.3	12.6	12

### Transcriptional profiling reveals distinct gene expression patterns associated with site and between control and IBD derived organoids

**Fig. 2:**
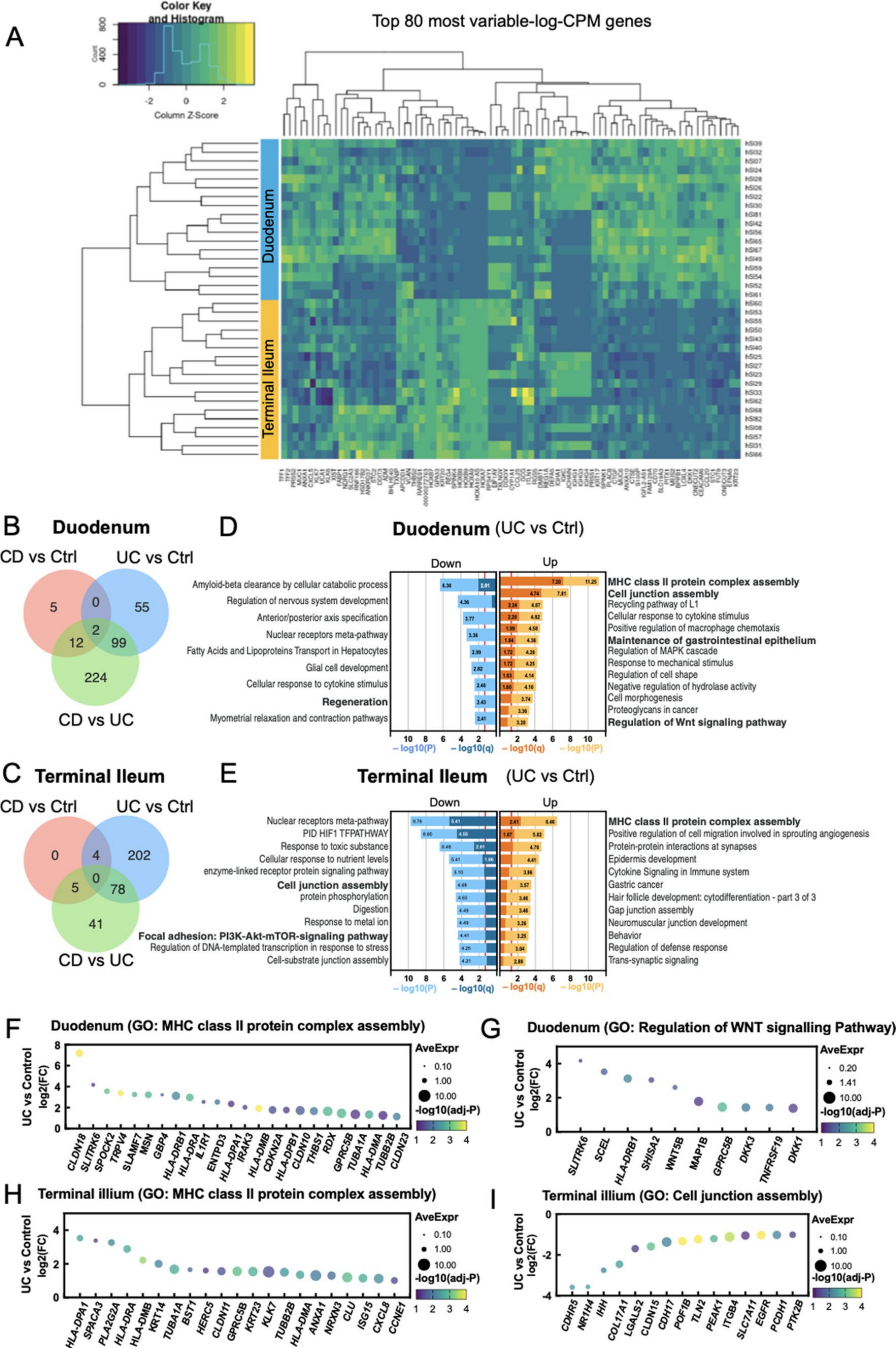
Transcriptional differences are observed between control- and IBD-derived organoids. (**A**) Heat map of normalised expression levels of the top 80 most variable genes amongst duodenum and terminal ileum organoid lines. Blue bar indicates duodenum- derived organoids and orange bar denotes terminal ileum- derived organoids, *n* = 7 control, *n* = 7 CD, *n* = 4 UC patient samples, with a total of 36 patient-derived organoid lines for RNA-sequencing. Normalised CPM values are shown on a log_2_ scale. (**B** and **C**) Venn diagrams of the number of DEGs when comparing CD-, UC- and control-derived organoids from the duodenum (**B**) and terminal ileum (**C**). DEGs determined by applying false discovery rate (FDR) ≤ 0.05 and log fold change (log_2_FC) ≥ 1 or ≤ -1. (**D** and **E**) Functional gene annotation of significant DEGs between UC and control organoids from the duodenum (**D**) and terminal ileum (**E**). Gene ontology was carried out with the DEGs, where the resulting graphs, shown on a log_10_ scale, display the significant values of enriched gene ontologies after analysis in Metascape^31^. (**F**–**I**) Dot plot showing the average and differential expression values of genes belonging to over-represented gene ontology sets in duodenal-derived organoids for “MHC class II protein complex assembly” (**F**) and “regulation of WNT signalling pathway” (**G**), and in terminal ileum-derived organoids, “MHC class II protein complex assembly” (**H**) and “cell junction assembly” (**I**).

A subset of organoid lines were selected to evaluate the transcriptional differences between control and IBD-derived HIOs, where RNA-sequencing was performed on 36 organoid lines. These HIOs were derived from the duodenum and terminal ileum of 18 patients, with and without IBD (Supplementary Table 2). Whilst sample numbers are small, the top 80 most variable genes expressed amongst these organoids showed that the major influences were due to the site from which the organoid was derived (Fig. 2A and supplementary Fig. 1A). Amongst the duodenal-derived organoids, 19 differentially expressed genes (DEGs) were identified between CD compared to control, 156 DEGs between UC and control and 337 DEGs between CD and UC, where there were two DEGs (*TRIB3* and *CLDN18*) shared amongst all the different comparisons (Fig. 2B, supplementary Fig. 1B and supplementary Table 3). Whilst no DEGs were shared between terminal ileum-derived organoids, nine DEGs were identified between CD and control, 284 DEGs between UC and control, and 124 DEGs between CD and UC (Fig. 2C, supplementary Fig. 1B and supplementary Table 3). Comparison of duodenum- and terminal ileum-derived organoids revealed 33 shared DEGs between UC and control organoids (supplementary Fig. 1C). Due to the low number of DEGs between CD and control organoids, the overlap in genes for these two anatomical locations was not carried out, additionally, gene ontology analysis was performed on DEGs from UC and control organoids only, where the upregulated genes showed significant enrichment for MHC class II protein complex assembly in both duodenum- and terminal ileum-derived UC organoids, when compared to control organoids (q-value ≤ 0.05, Fig. 2D and E). Average expression levels of annotated genes in this category are shown in Fig. 2F and H. Specific to the duodenum, upregulated genes showed significant enrichment for processes including cell junction assembly, cellular response to cytokine stimuli and maintenance of gastrointestinal epithelium (q ≤ 0.05) in UC-derived organoids (Fig. 2D). In addition, regulation of Wnt signalling pathway, a key signalling pathway for intestinal stem cell self-renewal, is also enriched in UC derived organoids (*q* = 0.124, Fig. 2D and G). In the terminal ileum-derived organoids, upregulated genes showed enrichment for cytokine signalling in the immune system (*q* = 0.081), whereas downregulated genes showed over-representation in cell junction assembly (*q* ≤ 0.05, Benjamini-Hochberg procedure, Fig. 2E and I).

### Patient-derived organoids reveal variable responses to patient-isolated bacteria

We hypothesised that differences in epithelial response may be observed following exposure to microbiome-derived bacterial isolates. To investigate this, a phylogenetic tree summarising the bacterial species cultured was generated (Supplementary Fig. 2A). Closely related bacterial isolates from a control patient and patient diagnosed with IBD were selected to study their influence on matched patient-derived organoid cultures (Supplementary Fig. 2).

Regions of the tree where a species was detected in a minimum of two control patients and two IBD patients was investigated further. The isolates in each clade were visualised in subsequent phylogenetic tree generations to observe whether the isolates grouped together based on patient diagnosis and/or tissue inflammatory status; representative isolates were then selected from each clade (Supplementary Fig. 2B and E). The first clade had isolates from the *Bacteroidaceae* family, where a representative isolate from a control (isolate named CC01454) and an IBD (CC01453) patient were selected. The second clade investigated had isolates from the *Enterobacteriaceae* family, where a representative isolate from a control (CC00517) patient and IBD (CC00518) patient were selected. These isolates were tested for their ability to either tolerate or grow in aerobic conditions (Supplementary Fig. 2C, D, F and G). All four representative isolates were grown in anaerobic conditions but delivered to HIOs via microinjection under aerobic conditions, where the control-associated bacterium was microinjected into its matched control-derived (terminal ileum) organoid line and the IBD-associated bacterium was microinjected into its matched organoid line, and vice versa, to study the bacterium’s effect on human intestinal epithelial cells.

**Fig. 3:**
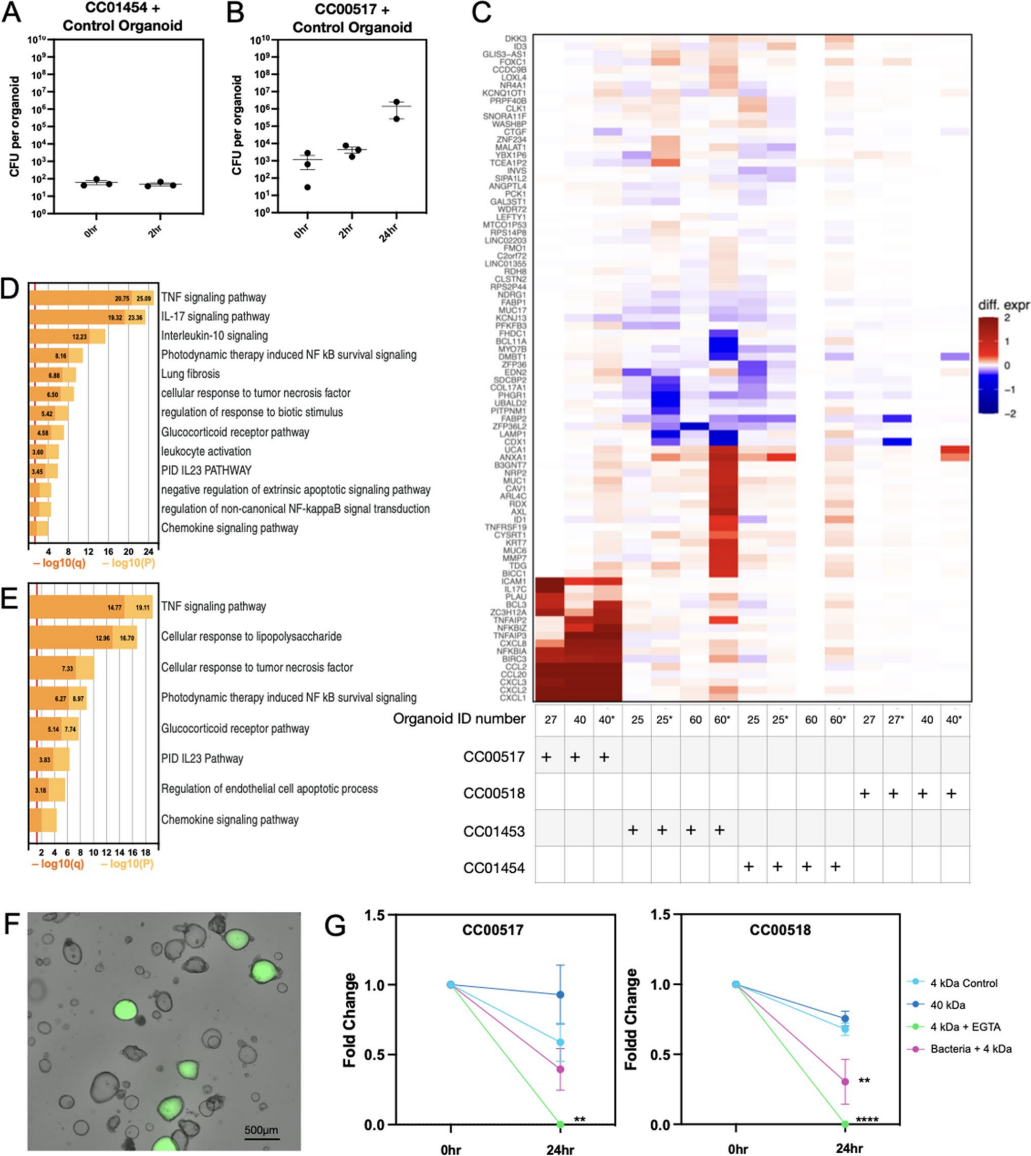
Response to patient-associated bacteria is strain-specific. (**A**) CFU per organoid following microinjection of control organoids with CC01454. (**B**) CFU per organoid following microinjection of control organoids with CC00517. (**A** and **B**) CFU per organoid was calculated from the average of 4 microinjected organoids pooled for quantification. Graphs are shown on a log scale (*n* = 3 experimental repeats, mean ± SEM plotted). Mann- Whitney U- test, no significance detected. (**C**) Heat map of 86 DEGs following microinjection of control or IBD derived organoids with bacterial isolates CC00517, CC00518, CC01453 or CC01454 (*n* = 3 experimental repeats, with 45–50 organoids picked per condition for RNA-sequencing). Genes with FDR < 0.05 and absolute shrunken fold-change (VST transformation in DESeq2^42^> 1.0 in one or more contrasts were included. Colour scale is differential gene expression (shrunken log_2_ fold change). Numbers underneath the heatmap indicates the organoid line (ID number) that was microinjected. * indicates organoids microinjected with bacteria compared to its corresponding exposure control organoids, all other comparisons are to mock-injected control organoids. (**D**) Gene ontology analysis of 18 upregulated DEGs following microinjection of control-derived organoids (line hSI27) with isolate CC00517. DEGs determined by applying false discovery rate (FDR) ≤ 0.05 and log fold change (log_2_FC) ≥ 1 or ≤ – 1. (**E**) Gene ontology analysis of 13 upregulated DEGs following microinjection of IBD-derived organoids (line hSI40) with isolate CC00517. (**F**) Representative image of organoids microinjected with bacteria and 4 kDa FITC-dextran. Scale bar 500 μm. (**G**) Change in fluorescence following microinjection of control organoids with 4 kDa FITC-dextran only (in cyan), 40 kDa FITC-dextran (in blue), 4 kDa FITC-dextran with EGTA treatment at 23 h timepoint (in green) or microinjection with isolate CC00517 or CC00518 (in pink). Statistical analysis carried out at 24 h timepoint, comparing conditions to the 4 kDa FITC-dextran microinjected control (*n* = 3 experimental repeats, where 20–25 organoids were microinjected per condition allowing 2–8 organoids to be analysed per condition with ImageXpress Pico Automated Cell Imaging System (Molecular Devices), mean ± SEM). Two-way ANOVA with Dunnett’s multiple comparison test (**p < 0.01, ****p < 0.0001).

Isolates CC01453 and CC01454 (from the *Bacteroidaceae* family) have an average nucleotide identity (ANI) of 97.9% and are anaerobic microbes yet withstand exposure to aerobic conditions (Supplementary Fig. 2C and D), showed delivery of viable bacterial loads pre-, mid-, and post-microinjection (3.89 × 10^1^ to 1.14 × 10^3^ CFU per injection, Supplementary Fig. 3A). Isolates CC00517 and CC00518 (from the *Enterobacteriaceae* family) have a 96.96% ANI score, can grow in aerobic environments (Supplementary Fig. 2F and G), and delivery of viable bacteria pre-, mid-, and post-microinjection was also detected (5.27 × 10^2^ to 4.08 × 10^5^ per injection, Supplementary Fig. 4A). For all procedures, bacteria were successfully microinjected into the lumen of organoids, where the bacterial load within the organoid was quantified at the 0 h (132.0 ± 32.4 CFU per organoid for CC01454 and CC01453, 2082 ± 426.8 CFU per organoid for CC00517 and CC00518) and 2 hr timepoint (146.9 ± 43.2 to 1818.3 ± 605.9 CFU per organoid for CC00517 and CC00518, Fig. 3A and B and Supplementary Figs. 3B and 4B). During microinjection, bacteria were detected in the culturing medium. These organoids exposed to bacteria in the media were collected allowing for comparison with microinjected organoids (Supplementary Fig. 3C and 4 C). Mock injected organoids, organoids microinjected with bacterium, and organoids exposed to bacterium in the culturing medium were collected 2 hrs following the introduction of bacteria for transcriptional profiling.

Transcriptional analysis of organoids 2 h following microinjection with CC01453 compared to its mock injected control resulted in no significant DEGs (FDR ≤ 0.05 and log_2_FC ≥ 1 or ≤ -1, Supplementary Fig. 5). Two DEGs were detected when CC01454 was microinjected into its corresponding control organoid line (*BCL11A* and *GUCY2C*), and 50 DEGs were detected when it was microinjected into an IBD organoid line (Supplementary Fig. 5). Gene ontology of these 50 genes resulted in no significant enrichments. As no clear pattern of transcriptional changes was observed when microinjecting isolates from the *Bacteroidaceae* family into organoids, when compared to mock injected controls, this was not investigated further (Fig. 3C and Supplementary Fig. 6).

When studying the effect of isolates from the *Enterobacteriaceae* family, both control and IBD derived organoids showed an upregulation of immune signalling genes, such as *CXCL1*, *CXCL2*, *CXCL3* and *NFKBIA*, in response to isolate CC00517, but not isolate CC00518 (Fig. 3C and Supplementary Fig. 6). This response was only observed when the control *Enterobacteriaceae* isolate was microinjected into the organoid lumen, enabling contact with the apical surface of the epithelial cells, and not when the basolateral surface was exposed to the bacterium in the media (Supplementary Fig. 6). Gene ontology analysis of the 18 significantly upregulated DEGs following microinjection of CC00517 into control derived organoids (supplementary Fig. 7) revealed enrichment for TNFα (q ≤ 0.05) and IL-17 signalling (q ≤ 0.05, Fig. 3D), where the top three genes with the greatest log fold change were *IL17C* (log_2_FC = 4.27), *ICAM1* (log_2_FC = 4.19) and *CCL20* (log_2_FC = 3.99). Similarly, the 13 significantly upregulated DEGs identified following microinjection of CC00517 into IBD derived organoids (supplementary Fig. 7) revealed enrichment for TNF signalling pathway (q ≤ 0.05, Fig. 3E), where the top three genes with the greatest log fold change were *CXCL2* (log_2_FC = 3.49), *CXCL1* (log_2_FC = 3.07) and *CCL2* (log_2_FC = 2.69).

Due to the inflammatory signature observed in response to the control *Enterobacteriaceae* isolate, the potential effect on epithelial permeability was investigated at a later (24 h) timepoint. This revealed that luminal bacteria were detectable and had replicated within the organoids’ lumen (Fig. 3B and Supplementary Fig. 4). Microinjection with or without CC00517 and CC00518 in addition to 4 kDa FITC-dextran into the control organoid line was used to assess epithelial permeability, by quantifying the organoids that retained a luminal fluorescent signal at 24 h (Fig. 3F and Supplementary Fig. 8). Organoids were microinjected with 40 kDa FITC-dextran, as a negative control, as molecules of this size cannot pass through the epithelial membrane. Treatment of 4 kDa FITC-dextran microinjected organoids with 3 mM EGTA, to disrupt adherens junctions was included as a positive control, where no luminal fluorescent signal was detected at endpoint, as expected (Fig. 3G and Supplementary Fig. 8).

Microinjection of control organoids with CC00517 showed no significant differences when compared to 4 kDa mock-injected organoids at the 24 h timepoint (Fig. 3G). However, the microinjection of control organoids with CC00518 showed a loss in luminal fluorescent intensity at the 24 h timepoint, compared to the 4 kDa microinjected control (Fig. 3G, pink point vs. cyan point at 24 h timepoint, *p* = 0.0015, Dunnett’s multiple comparisons test), suggesting that this isolate can increase epithelial permeability.

## Discussion

This study demonstrates a novel, patient- and site- specific method of interrogating host-microbe interactions. Here, we have demonstrated this with a precision, although labour-intensive technique to interrogate a small number of these specific interactions. No differences in organoid establishment were observed between control and IBD samples from the duodenum or terminal ileum biopsies, a result in line with findings from Dotti et al.^9^, Noben et al.^43^, Howell et al.^13^ and Dennison et al.^44^, where these studies used a mix of ileum and colon samples for organoid generation. In this study, organoids were isolated from the small intestine. This allowed analysis of more proximal sites of antigenic interaction, often distant to the site of inflammation, which may confound detailed enquiry.

Indeed, transcriptional profiling of a subset of organoid lines revealed that samples clustered based on intestinal region rather than patient diagnosis and demonstrated transcriptional changes in the duodenum, a site that is not typically associated with IBD. This data further supports the findings of Lim et al. (2014), where their study identified increased epithelial shedding and leakage in the duodenum of IBD patients^45^. Though the focus of their study was on epithelial permeability in the duodenum, it supports the notion that proximal regions can exhibit disease-associated changes, despite not being detected by clinical screening procedures such as endoscopy and histology^45^. Looking specifically at duodenum- or terminal ileum-derived organoids, it was interesting to observe that samples do not cluster based on patient diagnosis, a result also observed by Howell et al.. (2018)^13^. Amongst the duodenum derived organoids, we identified two DEGs that are shared amongst the pairwise conditions (Fig. 2B), TRIB3 belongs to the tribbles pseudokinase family and is induced in response to ER stress^46^, can modulate MAPK signalling^47^, and has been shown to activate the expression of proinflammatory cytokines in some contexts^48^. These pathways are all implicated in the pathogenesis of IBD^49,50^. CLDN18 is a tight junction marker which are highly disrupted with barrier dysfunction in IBD. Dysregulation of a number of claudin family members have been identified in IBD^51^. This includes CLDN18 which is elevated in colitis^52^. Further interrogation of the transcriptional data shows that there are some specific differences in transcriptional profiles between patient disease type. CD-derived organoids show a modest difference in both intestinal regions. Whilst UC presents in the colon of patients, this study demonstrated that proximal sites can still exhibit transcriptional changes, where duodenal- and terminal ileum-derived organoids from patients with UC exhibit an upregulation in HLA-related transcripts (such as *HLA-DRA* and *HLA-DRB1*) when compared to control (Fig. 2F and H), a finding that is also observed in IBD-derived colonoids studied by Kelsen et al. (2021)^53^. The upregulation of these genes indicates the presence of adaptive immune response signalling within the UC-derived organoid lines. Additionally, a comparison of UC- and control-derived organoids from these two sites revealed 33 overlapping DEGs, further indicating that transcriptional changes can still be exhibited in proximal intestinal sites, however, there are a large number of DEGs that do not overlap, this is unsurprising due to the inherent differences between the duodenum and terminal ileum, and our observation that organoid derived lines clustered according to tissue site. Comparing CD to UC organoids, it was observed that the two IBD-related diseases varied at the transcriptional level. These differences could be due to the involvement of different tissue layers in disease where CD can involve the full thickness of the intestinal layers, whereas UC involves inflammation of the mucosa in the colon and rectum^54^. This study utilised organoid cultures comprised of epithelial cells only, allowing the investigation of bacterium directly on intestinal epithelial cells. These cultures do not include other host cell types, such as immune cells and mesenchymal cells, which are known to influence intestinal epithelium and can be added in a co-culture system^55,56^. Our system offers future potential to incorporate these additional components to more accurately model the microbe-epithelium-immune interactions. Perhaps without the interaction of these cell types in culture, signalling cues and crosstalk of cells that are not maintained in vitro, results in aspects of disease not being reflected at the transcriptional level in organoids. Furthermore, Lee et al.^57^ observed that with later passages (passage ≥6), CD-derived organoids exhibited no significant differences in cell viability and wound healing, when compared to control-derived organoids, unless treated with TNFα. These studies suggest inflammatory cues may be required to observe differences in epithelial response ex vivo. Additionally, taking into consideration the loss of phenotype observed at passages ≥6, by Lee et al.^57^, it is possible that variations in organoid passage number could confound the RNA-sequencing data here. Whilst RNA was collected prior to passage 6 for most organoid lines for RNA-sequencing, there was one CD-derived organoid line where RNA was collected at passage 6, a passage at which the disease phenotype may have resolved. While these differences are interesting, especially given the proximal nature of the samples, the small numbers analysed make any conclusions speculative.

Through microinjection of HIOs, an inflammatory response was observed within both control and IBD-derived organoid lines when exposed to bacterial isolate CC00517, compared to CC00518. TNF and IL-17 signalling were activated. These pathways have been implicated in cell death, regulation of inflammation, host defence and tissue repair^58,59^. Given the upregulation was from the control-derived bacteria, this result may seem initially surprising. However, an inflammatory response is not always deleterious. Intestinal epithelial cells sense gut commensals, which leads to the initiation of an immune response^60^, albeit different to the immune signalling activation in response to pathogenic bacteria^60^. Indeed, in the case of a pathogenic bacterial infection, inflammation is of course necessary and required for the infection to be cleared effectively^61^. It has been hypothesised that in IBD, it is a failure of an appropriate response to microbial antigens that leads to the uncontrolled inflammation^62^. This project studied closely related bacteria in isolation to better understand mechanistic differences in epithelial response. In patients, the microbiome exists in an ecosystem, where it is conceivable that closely related species may fill a niche that contributes to either an inflammatory or homeostatic response.

Here, we show an in-depth study into epithelial responses to a subset of patient microbiome isolates. Importantly, the microinjection technique, while labour intensive, allows for comprehensive investigations of individual bacterial isolates and their specific effects on patient intestinal epithelial cells. Whilst this technique allows delivery of bacteria to the apical surface of intestinal epithelial cells, it is difficult to control for the overgrowth of bacteria in the media and exposure to the basolateral surface. To circumvent this, we analysed organoids that were exposed to bacteria in the media as a control, however, this does present with limitations such as differences in bacterial load which could have differential effects on epithelial and transcriptional responses. Microinjection allows delivery of microbes into a low O_2_ environment^63,64^, thus representing a physiologically relevant model of host-microbe interactions. With the co-injection of bacterial isolates with 4 kDa FITC-dextran, epithelial permeability could be assessed. Whilst an increase in epithelial permeability was observed with CC00518, no significant differences was observed with CC00517. These results may be confounded by the variation in response of the control organoids in this experiment. In this study, we analysed 4 bacterial isolates and their effect on a sub-set of patient derived organoids. While this demonstrated some interesting results, the variation of the effect itself suggests a high degree of heterogeneity in host response to commensal organisms. Given the significant labour-intensive method of microinjection, and the vast numbers of potential host-microbe interactions, future studies should perhaps focus on more scalable techniques, such as the growth of organoids on filters in 2D, to understand these complex interactions. As well as only a small number of strains, and limited patient sample size, these experiments only included oxygen tolerant species, where future work would have to include protocols that allow analysis in appropriately anaerobic conditions.

The unique biobank of paediatric intestinal organoids with molecular profiling and associated bacteria generated has allowed interrogation of the epithelium and its response to stimuli that are both patient- and site-specific. This study has shown that the “inflammatory” signal in epithelium can be derived from commensal organisms and that bacteria from IBD patients can potentially lead to epithelial permeability. This variability in response at both a host and bacterial level demonstrates the importance of such a biobank, with future studies needing a higher-throughput model for experiments from this and other resources. Such a technique may be used in developing microbiome-based therapeutics for IBD, for which interest is increasing. This study demonstrates a model of interrogating site-specific microbiome-epithelial interactions and their disruption in IBD.

## Supplementary Information

Below is the link to the electronic supplementary material.

Supplementary Material 1

Supplementary Material 2

Supplementary Material 3

Supplementary Material 4

## Data Availability

Organoid cultures are available upon request through the Monash Organoid Program.Purified isolates have been deposited in the Australian Microbiome Culture Collection (AMCC) (https:/ausmicc.org.au). Transcriptional data deposited in GEO (GSE288179) (https:/www.ncbi.nlm.nih.gov/geo/query/acc.cgi?acc=GSE288179).

## Abbreviations

ANI: Average nucleotide identity
CFU: Colony forming units
CD: Crohn’s disease
DEGs: Differentially expressed genes
EGTA: Ethylene glycol- *bis*(β-aminoethyl)-N, N, Nʹ, Nʹ-tetra acetic Acid
FDR: False discovery rate
GIT: Gastrointestinal tract
IBD: Inflammatory bowel disease
PBS: Phosphate buffered saline
UC: Ulcerative colitis
YCFA: Yeast casitone fatty acids
