# Supplementary material for: Patient-derived intestinal organoids as a model for site-specific mucosal bacterial interactions in paediatric inflammatory bowel disease
Source: Sci Rep. 2026 Apr 1;16:15359. doi: 10.1038/s41598-026-46184-8 (PMC13184080; doi:10.1038/s41598-026-46184-8)
Supplement: Supplementary file 1 — Supplementary Material 1 [file 41598_2026_46184_MOESM1_ESM.pdf]

Patient-derived intestinal organoids as a model for site-specific mucosal bacterial interactions in paediatric inflammatory bowel disease.

Eva Chan, PhD,[1,2,3,4] Wing Hei Chan, PhD,[1,2] Genevieve Kerr, PhD[1,2] Stuart K. Archer, PhD,[1,2,5] Thierry Jardé, PhD,[1,2] Rebekah M. Engel, PhD,[1,2,6] Jodee A. Gould, BSc (Hons),[3,4] Shanika L. Amarasinghe, PhD,[1,2] Emily L. Rutten, BSc (Hons), [3,4] Gemma L. D'Adamo, MD PhD,[3,4] Emily L. Gulliver, PhD,[3,4] Linden J. Gearing, PhD,[3,4] Samuel C. Forster, PhD[3,4*] Edward M. Giles, MBBS PhD,[3,4,7*], Helen E. Abud, PhD,[1,2,6*]

[1]Department of Anatomy and Developmental Biology, Monash University, Clayton, VIC 3800, Australia

[2]Development and Stem Cells Program, Monash Biomedicine Discovery Institute, Clayton, VIC 3800, Australia

[3]Centre for Innate Immunity and Infectious Diseases, Hudson Institute of Medical Research, Clayton, VIC 3168, Australia

[4]Department of Molecular and Translational Sciences, Monash University, Clayton, VIC 3800, Australia

[5] Monash Genomics and Bioinformatics Platform, Monash University, Clayton, VIC 3800, Australia

[6]Cabrini Monash University Department of Surgery, Cabrini Hospital, Malvern VIC 3144, Australia

[7]Department of Paediatrics, Monash University, Clayton, VIC 3800, Australia

Correspondence to: Professor Helen E. Abud, Monash Biomedicine Discovery Institute, Monash University, Clayton Victoria, Australia helen.abud@monash.edu. Associate Professor Samuel C Forster, Hudson Institute of Medical Research, Clayton, Victoria, Australia sam.forster@hudson.org.au, Dr Edward M Giles, Hudson Institute of Medical Research, Clayton, Victoria, edward.giles@monash.edu

EMG, SCF and HEA contributed equally and are joint senior authors.

SUPPLEMENTARY FIGURES

Supplementary Figure 1: Transcriptional analysis of patient-derived duodenal and terminal ileum organoids from patients with or without IBD.
(A) PCA plot of organoids from control and IBD patients. Duodenal organoids are depicted in red and terminal ileums in blue. Further information on patient-derived lines are available in Supplementary Table 2. (B) Visualisation of differential gene expression by plotting $-\log_{10}$(FDR) against shrunken $\log_2$(fold change) on volcano plots. Contrasts plotted are Crohn's vs control, Crohn's vs Ulcerative Colitis and Ulcerative Colitis vs Control, from the duodenum and terminal ileum. Red dotted line indicates FDR=0.05. Genes labelled on volcano plots were selected based on a ranking metric which was generated from the product of absolute $\log_2$(fold change) and $-\log_{10}$(FDR), then the top-ranked significant named genes, up to a maximum of 20 genes, were labelled. n=7 Crohn's disease organoids, n=7 control organoids and n=4 ulcerative colitis organoids, per site. (C) Venn diagram to show the number of DEGs that overlap when comparing UC- and control-derived organoids from the duodenum and terminal ileum.

Supplementary Figure 2: Identification of bacterial candidates associated with control and newly diagnosed IBD patients.
(A) Shared species amongst IBD and control patients. (B) Phylogenetic relationship of a subset of *Bacteroidaceae* bacterial isolates from the region outlined with the light grey box of (A). (C) Growth of anaerobic bacterial isolate CC01454 (isolated from control patient) following exposure to aerobic conditions. (D) Growth of anaerobic bacterial isolate CC01453 (isolated from patient with IBD) following exposure to aerobic conditions. (C and D) Values plotted on a log scale (n=3 experimental repeats, mean $\pm$ SD). Mann- Whitney U-test, no significance detected. (E) Phylogenetic relationship of a subset of *Enterobacteriaceae* bacterial isolates from the region outlined with the dark grey box in (A). (F) Growth of bacterial isolate CC00517 (isolated from control patient) in aerobic conditions over time. (G) Growth of bacterial isolate CC00518 (isolated from IBD patient) in aerobic conditions over time. (F and G) n=3 experimental repeats, mean $\pm$ SD.

Supplementary Figure 3: CFU from microinjection of control (line hSI60) or IBD (line hSI25) derived organoids with isolates from the *Bacteroidaceae* family, CC01453 or CC01454.
(A) Number of bacteria delivered per microinjection. (B) Number of bacteria detected within the organoid lumen at 0 hr and 2 hr following microinjection. (C) Number of bacteria detected in the surrounding media following microinjection of organoids.
Graphs are shown on a log scale. n=3 experimental repeats. Mean ± SEM plotted. Dotted line at 1.43 denotes the lower detection threshold. Mann- Whitney U- test, no significance detected.

Supplementary Figure 4: CFU from microinjection of control (line hSI27) or IBD (line hSI40) derived organoids with isolates from the *Enterobacteriacaea* family, CC00517 or CC00518.
(A) Number of bacteria delivered per microinjection. (B) Number of bacteria detected within the organoid lumen at 0 hr and 2 hr following microinjection. (C) Number of bacteria detected in the surrounding media following microinjection of organoids.
Graphs are shown on a log scale (n=3 experimental repeats, mean ± SEM plotted). Dashed line at 8.75e+009 denotes the upper detection threshold. Dotted line at 1.43 denotes the lower detection threshold. Mann- Whitney U- test, no significance detected.

Supplementary Figure 5: Transcriptional analysis of organoids microinjected with CC01453 and CC01454.
Visualisation of differential gene expression by plotting $-\log_{10}$(FDR) against shrunken $\log_2$(fold change) values on volcano plots. Plotted are human small intestinal organoid lines hSI25 and hSI60 microinjected with bacterial isolates (CC01453 or CC01454) compared to mock-injected or exposed to bacteria controls. Red dotted line indicates FDR=0.05. Genes labelled on volcano plots were selected based on a ranking metric which was generated from the product of absolute $\log_2$(fold change) and $-\log_{10}$(FDR), then the top-ranked significant named genes, up to a maximum of 20 genes, were labelled. n=3 experimental repeats.

Supplementary Figure 6: Heat map of DEGs following microinjection or exposure of control or IBD derived organoids with bacterial isolates CC00517, CC00518, CC01453 or CC01454 (n=3 experimental repeats for all conditions). Genes with FDR < 0.05 and absolute shrunken fold change (VST transformation in DESeq2 [1]) > 0.5 in one or more contrasts were included. Colour scale is differential gene expression (shrunken $\log_2$ fold change). Numbers underneath the heatmap indicates the organoid line (ID number) that was microinjected. Microinjected organoids were compared to mock-injected organoids, where * indicates organoids microinjected with bacteria compared to its corresponding organoids exposed to bacteria condition. Organoids exposed to bacteria were compared to mock-injected control organoids.

Supplementary Figure 7: Transcriptional analysis of organoids microinjected with CC00517 and CC00518.
Visualisation of differential gene expression by plotting $-\log_{10}$(FDR) against shrunken $\log_2$(fold change) values on volcano plots. Plotted are human small intestinal organoid lines hSI27 and hSI40 microinjected with bacterial isolates (CC00517 or CC00518) compared to mock-injected or exposed to bacteria controls. Red dotted line indicates FDR=0.05. Genes labelled on volcano plots were selected based on a ranking metric which was generated from the product of absolute $\log_2$(fold change) and $-\log_{10}$(FDR), then the top-ranked significant named genes, up to a maximum of 20 genes, were labelled. n=3 experimental repeats.

Supplementary Figure 8: Proportion of organoids retaining a FITC-dextran signal 24 hrs following microinjection (n=3 experimental repeats, mean ± SEM plotted). All conditions were compared to 4 kDa control (mock injection control), Ordinary One-way ANOVA with Dunnett's multiple comparison test (*$p<0.05$, **$p<0.01$).

Supplementary Figure 1

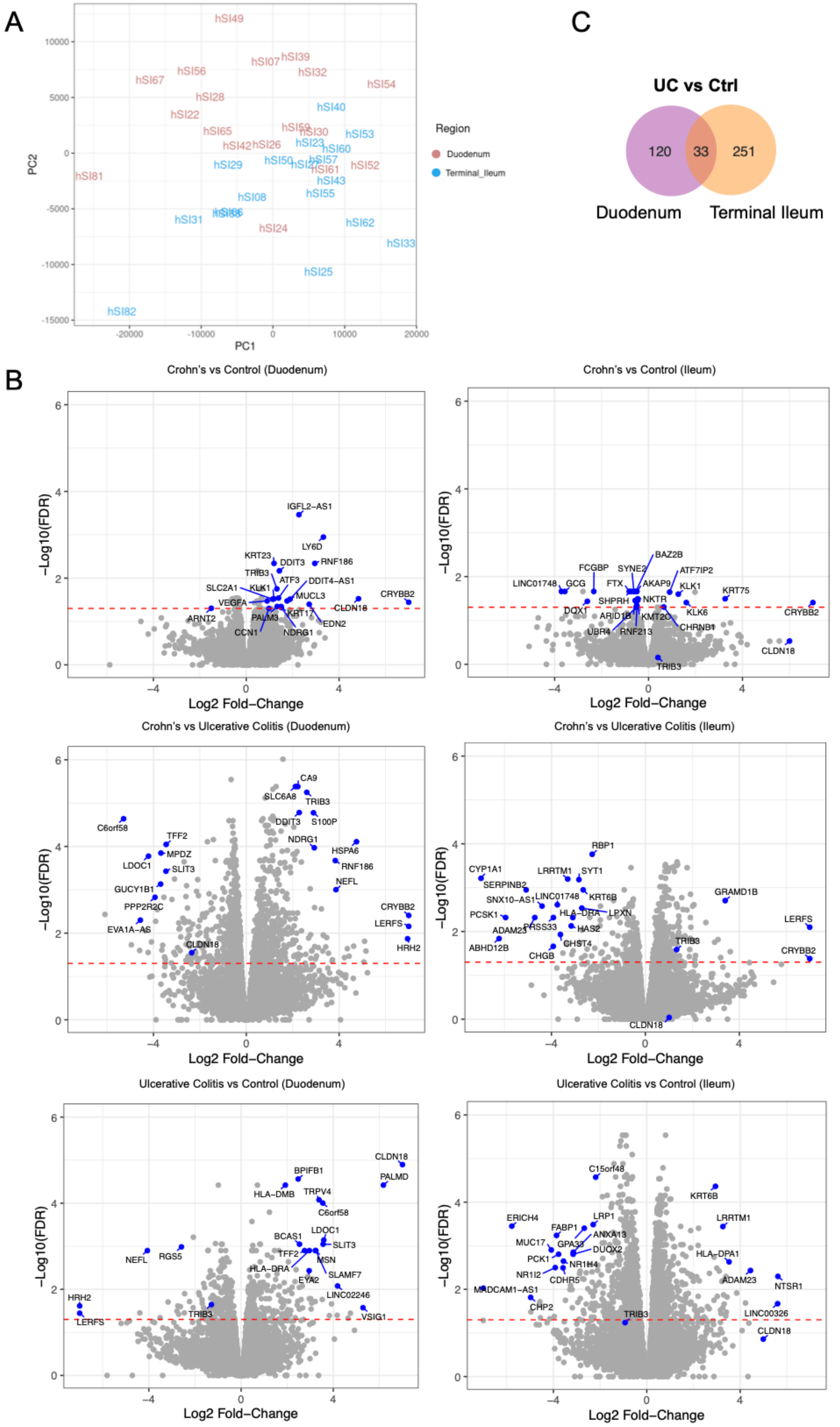

Supplementary Figure 2

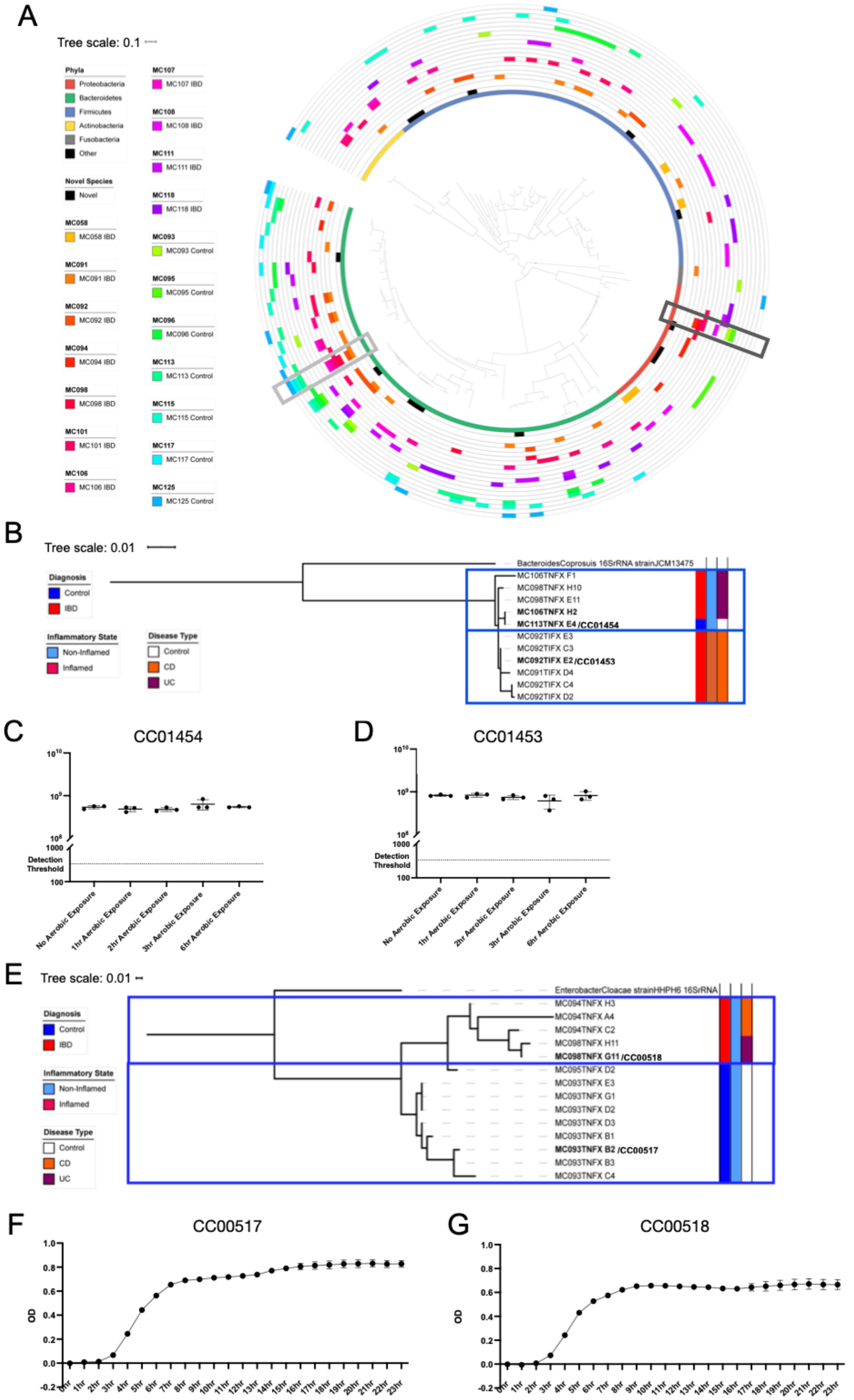

Supplementary Figure 3

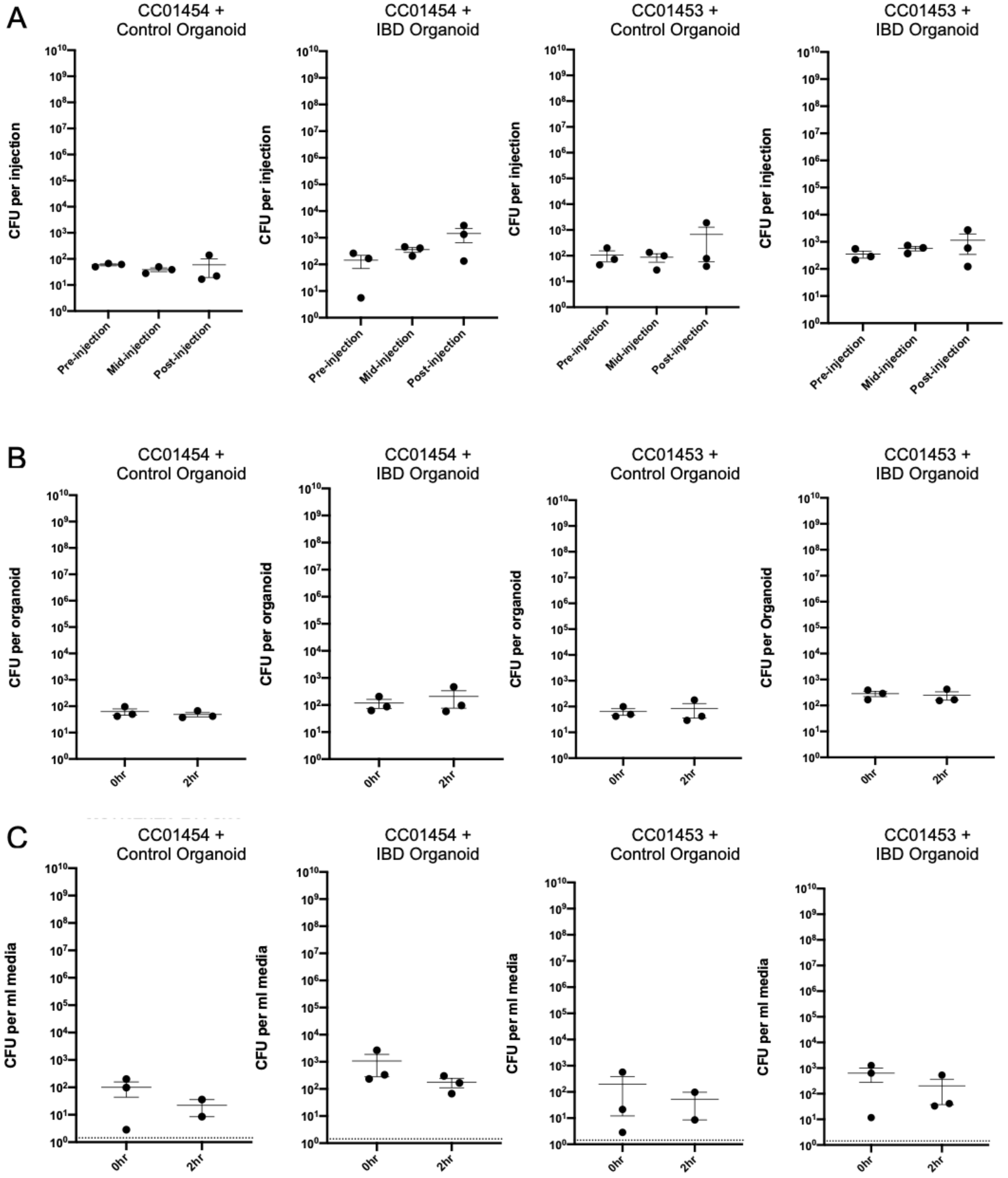

Supplementary Figure 4

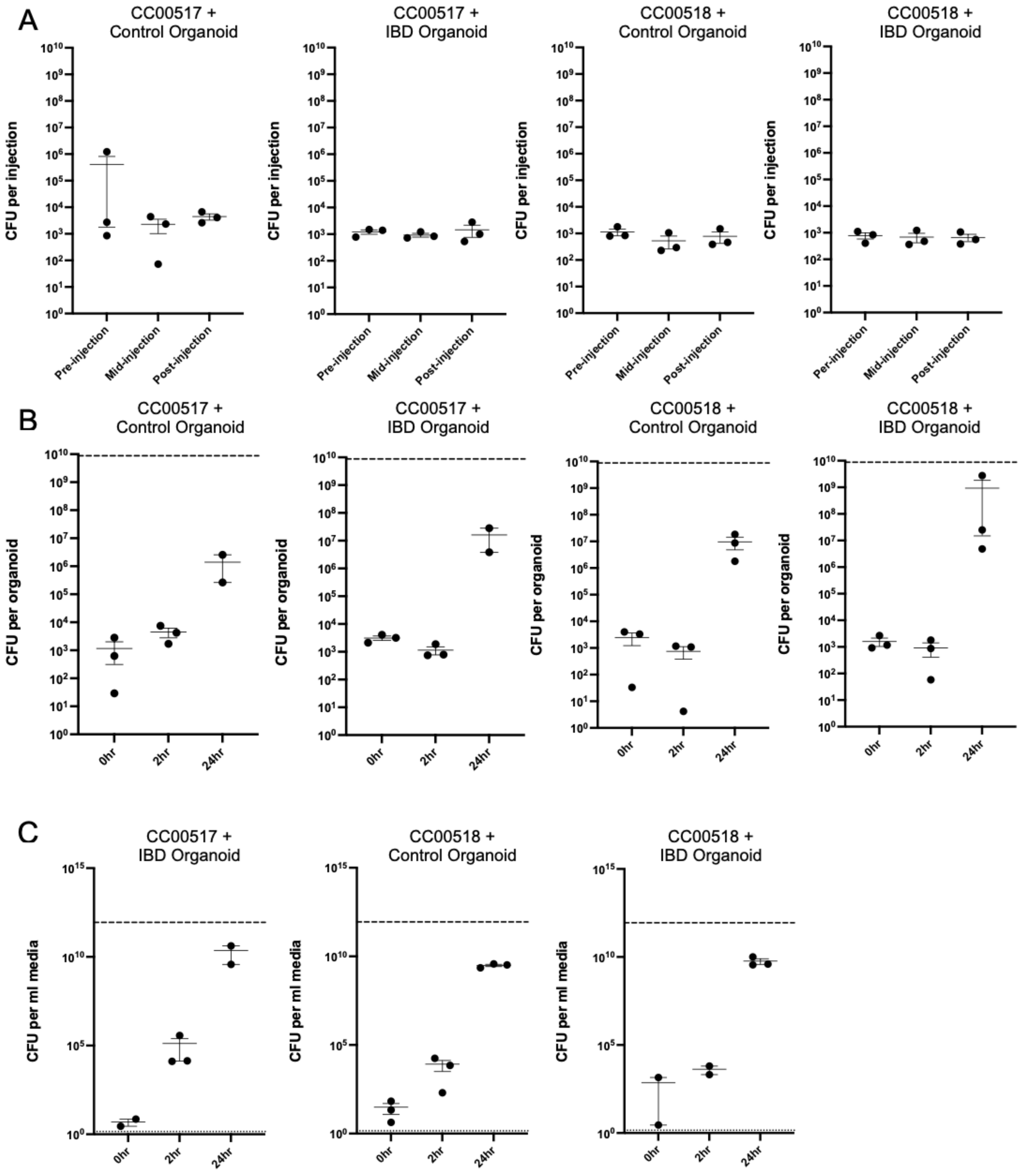

Supplementary Figure 5

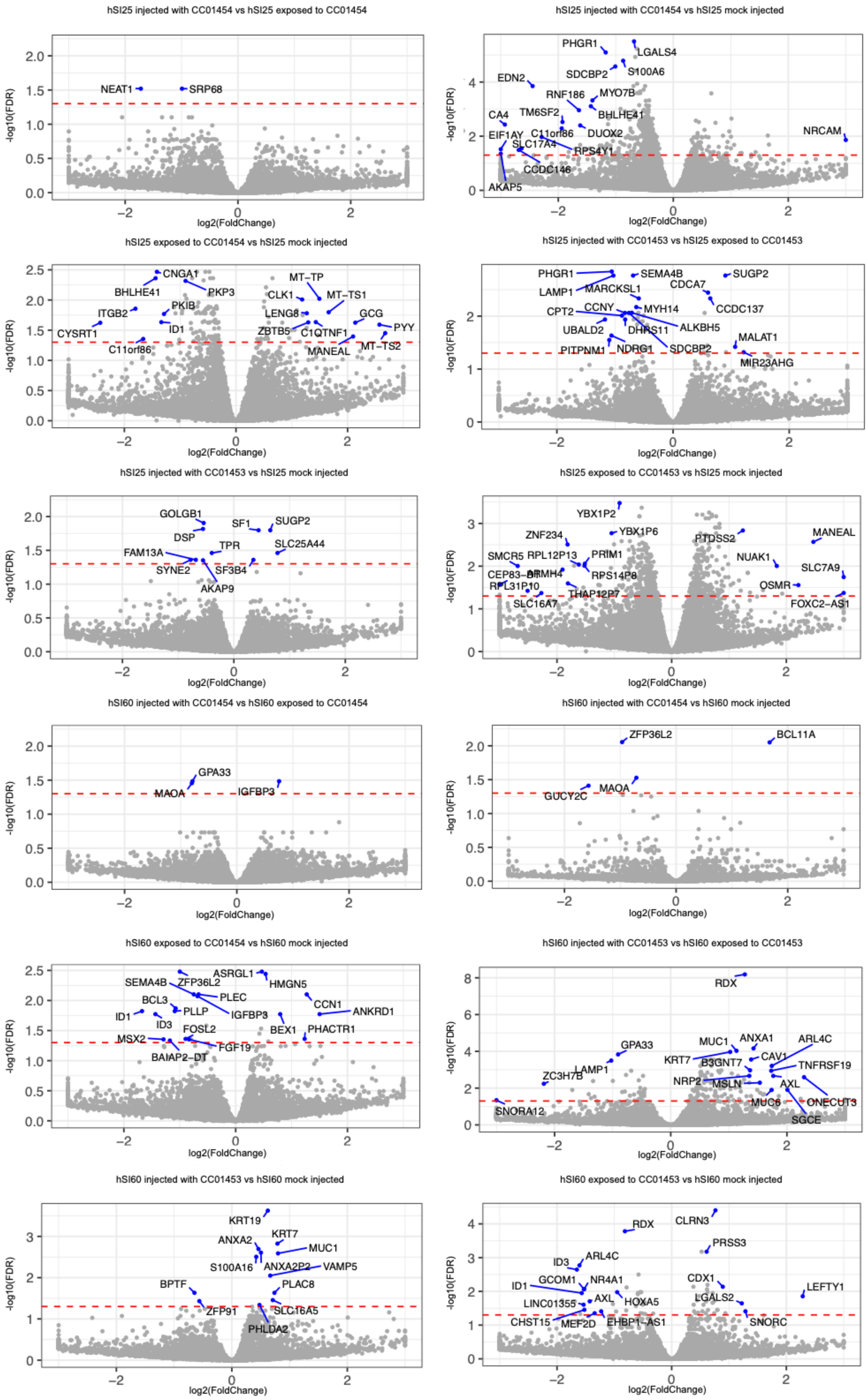

Supplementary Figure 6

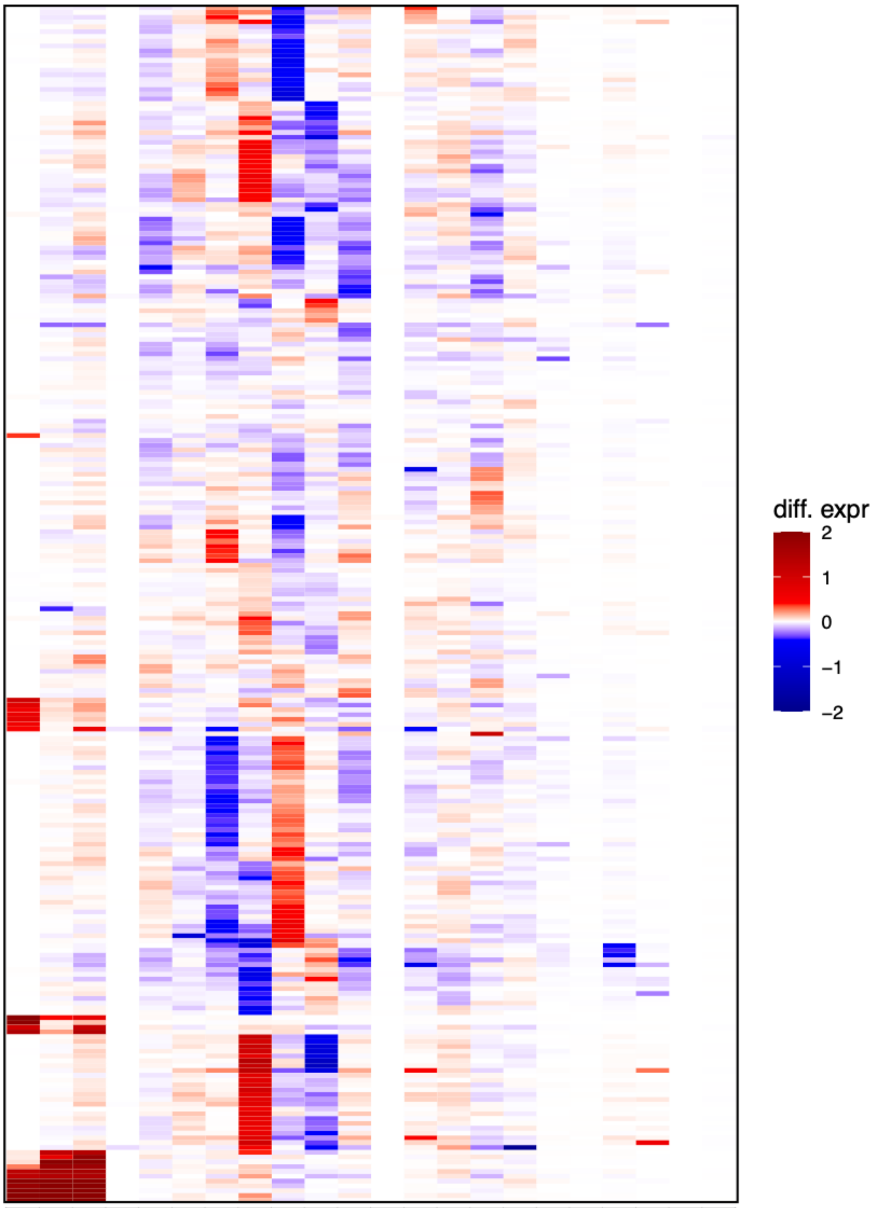

Organoid ID Number:	27	40	40 *	40	25	60	25 *	60 *	25	60	25	60	25 *	60 *	25	60	27	40	27 *	40 *	27	40
Microinjected with CC00517:	+	+	+																			
Microinjected with CC00518:																	+	+	+	+		
Microinjected with CC01453:					+	+	+	+														
Microinjected with CC01454:											+	+	+	+								
Exposed to CC00517:				+																		
Exposed to CC00518:																					+	+
Exposed to CC01453:									+	+												
Exposed to CC01454:															+	+						

Supplementary Figure 7

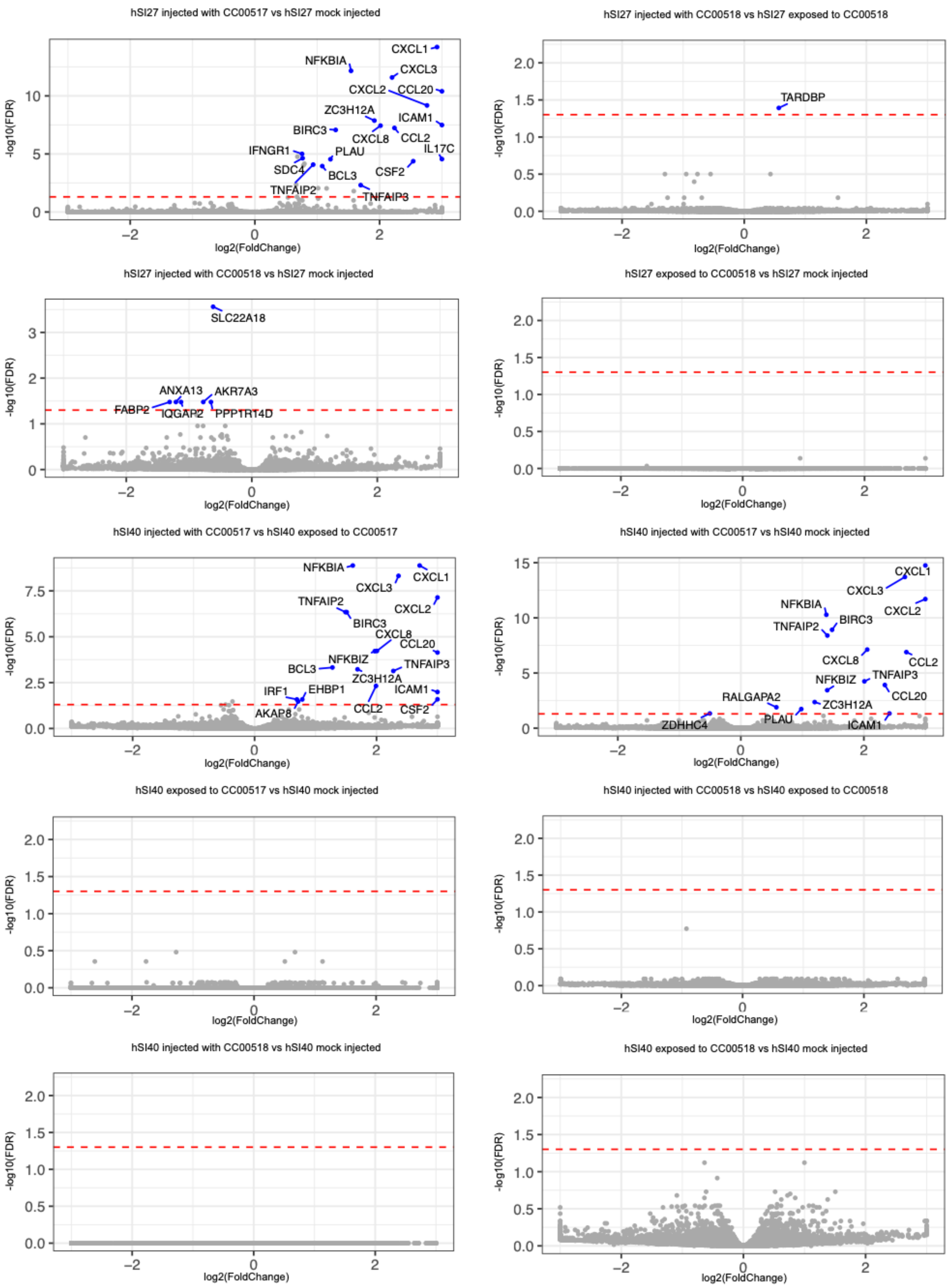

Supplementary Figure 8

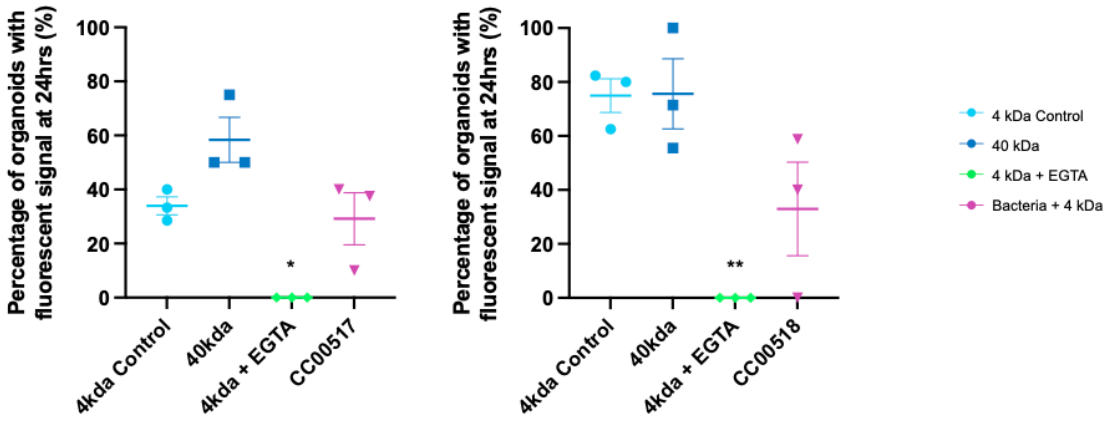

Supplementary Table 2: List of patient information, including diagnosis, tissue sample site, gender, age and pathology. “D” indicates Duodenum, “TI” indicates Terminal Ileum, “F” indicates Female, “M” indicates Male, “I” indicates Inflamed and “N” indicates Non-Inflamed. Two biopsies were collected per tissue site.

Patient	Organoid ID	Diagnosis	Biopsy Region	Gender	Bacterial Culture ID	Age (yrs)	Histology	Montreal Classification[1]
1	hSI07	New Crohn's	D	F	MC058	8	I	A1L3B1
	hSI08		TI				N	
2	hSI22	New Crohn's	D	M	MC091	8	N	A1L1B1
	hSI23		TI				I	
3	hSI24	New Crohn's	D	F	MC092	9	N	A1L1B1
	hSI25		TI				I	
4	hSI26	Control (scoped due to iron deficiency)	D	F	MC093	6	N	N/A
	hSI27		TI				N	
5	hSI28	New Crohn's	D	F	MC094	16	N	A1L4B1
	hSI29		TI				N	
6	hSI30	Control (scoped due to rectal bleeding)	D	M	MC095	16	N	N/A
	hSI31		TI				N	
7	hSI32	Control (diagnosed with IBS)	D	F	MC096	17	N	N/A
	hSI33		TI				N	
8	hSI39	New Ulcerative Colitis	D	F	MC098	12	N	A1E2S1
	hSI40		TI				N	
9	hSI42	New Crohn's	D	F	MC101	11	I	A1L3B1
	hSI43		TI				N	
10	hSI49	New Ulcerative Colitis	D	F	MC106	14	I	A1E3S2
	hSI50		TI				N	
11	hSI52	New Ulcerative Colitis	D	M	MC107	9	N	A1E3S3
	hSI53		TI				N	
12	hSI54	New Ulcerative Colitis	D	M	MC108	11	N	A1E3S3
	hSI55		TI				I	
13	hSI56	New Crohn's	D	F	MC111	15	N	A1L3B1
	hSI57		TI				I	
14	hSI59	Control	D	M	MC113	9	N	N/A
	hSI60		TI				N	
15	hSI61	Control (diagnosed with IBS)	D	F	MC115	17	N	N/A
	hSI62		TI				N	
16	hSI65	Control	D	M	MC117	12	N	N/A
	hSI66		TI				N	
17	hSI67	New Crohn's	D	F	MC118	13	I	A1L1B1
	hIS68		TI				I	
18	hSI81	Control (scoped due to iron deficiency)	D	F	MC125	7	N	N/A
	hSI82		TI				N	

Reference:

1. Satsangi J, Silverberg MS, Vermeire S, Colombel JF. The Montreal classification of inflammatory bowel disease: controversies, consensus, and implications. Gut. 2006;55(6):749-53.

Reference:

1. Love MI, Huber W, Anders S. Moderated estimation of fold change and dispersion for RNA-seq data with DESeq2. Genome Biol. 2014;15(12):550.
