# Supplementary material for: Patient-derived intestinal organoids as a model for site-specific mucosal bacterial interactions in paediatric inflammatory bowel disease
Source: Sci Rep. 2026 Apr 1;16:15359. doi: 10.1038/s41598-026-46184-8 (PMC13184080; doi:10.1038/s41598-026-46184-8)
Supplement: Supplementary file 3 — Supplementary Material 3 [file 41598_2026_46184_MOESM3_ESM.docx]

Supplementary Table 2: List of patient information, including diagnosis, tissue sample site, gender, age and pathology. “D” indicates Duodenum, “TI” indicates Terminal Ileum, “F” indicates Female, “M” indicates Male, “I” indicates Inflamed and “N” indicates Non-Inflamed. Two biopsies were collected per tissue site.

| Patient | Organoid ID | Diagnosis | Biopsy Region | Gender | Bacterial Culture ID | Age (yrs) | Histology | Montreal Classification[1] |
| --- | --- | --- | --- | --- | --- | --- | --- | --- |
| 1 | hSI07 | New Crohn's | D | F | MC058 | 8 | I | A1L3B1 |
|  | hSI08 |  | TI |  |  |  | N |  |
| 2 | hSI22 | New Crohn's | D | M | MC091 | 8 | N | A1L1B1 |
|  | hSI23 |  | TI |  |  |  | I |  |
| 3 | hSI24 | New Crohn's | D | F | MC092 | 9 | N | A1L1B1 |
|  | hSI25 |  | TI |  |  |  | I |  |
| 4 | hSI26 | Control (scoped due to iron deficiency) | D | F | MC093 | 6 | N | N/A |
|  | hSI27 |  | TI |  |  |  | N |  |
| 5 | hSI28 | New Crohn's | D | F | MC094 | 16 | N | A1L4B1 |
|  | hSI29 |  | TI |  |  |  | N |  |
| 6 | hSI30 | Control (scoped due to rectal bleeding) | D | M | MC095 | 16 | N | N/A |
|  | hSI31 |  | TI |  |  |  | N |  |
| 7 | hSI32 | Control (diagnosed with IBS) | D | F | MC096 | 17 | N | N/A |
|  | hSI33 |  | TI |  |  |  | N |  |
| 8 | hSI39 | New Ulcerative Colitis | D | F | MC098 | 12 | N | A1E2S1 |
|  | hSI40 |  | TI |  |  |  | N |  |
| 9 | hSI42 | New Crohn's | D | F | MC101 | 11 | I | A1L3B1 |
|  | hSI43 |  | TI |  |  |  | N |  |
| 10 | hSI49 | New Ulcerative Colitis | D | F | MC106 | 14 | I | A1E3S2 |
|  | hSI50 |  | TI |  |  |  | N |  |
| 11 | hSI52 | New Ulcerative Colitis | D | M | MC107 | 9 | N | A1E3S3 |
|  | hSI53 |  | TI |  |  |  | N |  |
| 12 | hSI54 | New Ulcerative Colitis | D | M | MC108 | 11 | N | A1E3S3 |
|  | hSI55 |  | TI |  |  |  | I |  |
| 13 | hSI56 | New Crohn's | D | F | MC111 | 15 | N | A1L3B1 |
|  | hSI57 |  | TI |  |  |  | I |  |
| 14 | hSI59 | Control | D | M | MC113 | 9 | N | N/A |
|  | hSI60 |  | TI |  |  |  | N |  |
| 15 | hSI61 | Control (diagnosed with IBS) | D | F | MC115 | 17 | N | N/A |
|  | hSI62 |  | TI |  |  |  | N |  |
| 16 | hSI65 | Control | D | M | MC117 | 12 | N | N/A |
|  | hSI66 |  | TI |  |  |  | N |  |
| 17 | hSI67 | New Crohn's | D | F | MC118 | 13 | I | A1L1B1 |
|  | hIS68 |  | TI |  |  |  | I |  |
| 18 | hSI81 | Control (scoped due to iron deficiency) | D | F | MC125 | 7 | N | N/A |
|  | hSI82 |  | TI |  |  |  | N |  |

Reference:

1. Satsangi J, Silverberg MS, Vermeire S, Colombel JF. The Montreal classification of inflammatory bowel disease: controversies, consensus, and implications. Gut. 2006;55(6):749-53.
